# Dynamics of a fractional order mathematical model for COVID-19 epidemic

**DOI:** 10.1186/s13662-020-02873-w

**Published:** 2020-08-14

**Authors:** Zizhen Zhang, Anwar Zeb, Oluwaseun Francis Egbelowo, Vedat Suat Erturk

**Affiliations:** 1 School of Management Science and Engineering, Anhui University of Finance and Economics, Bengbu, 233030 China; 2 Department of Mathematics, COMSATS University Islamabad, Abbottabad Campus, Abbottabad, 22060 Khyber Pakhtunkhwa Pakistan; 3 Division of Clinical Pharmacology, Department of Medicine, University of Cape Town, Cape Town, South Africa; 4 Department of Mathematics, Faculty of Arts and Sciences, Ondokuz Mays University, 55139 Samsun, Turkey

**Keywords:** COVID-19 epidemic, Stability analysis, Adaptive predictor–corrector algorithm, Fractional differential equations, Numerical simulations

## Abstract

In this work, we formulate and analyze a new mathematical model for COVID-19 epidemic with isolated class in fractional order. This model is described by a system of fractional-order differential equations model and includes five classes, namely, *S* (susceptible class), *E* (exposed class), *I* (infected class), *Q* (isolated class), and *R* (recovered class). Dynamics and numerical approximations for the proposed fractional-order model are studied. Firstly, positivity and boundedness of the model are established. Secondly, the basic reproduction number of the model is calculated by using the next generation matrix approach. Then, asymptotic stability of the model is investigated. Lastly, we apply the adaptive predictor–corrector algorithm and fourth-order Runge–Kutta (RK4) method to simulate the proposed model. Consequently, a set of numerical simulations are performed to support the validity of the theoretical results. The numerical simulations indicate that there is a good agreement between theoretical results and numerical ones.

## Introduction

Mathematical models describing infectious diseases have an important role both in theory and practice (see, for example, [6–8, 18, 34]). The construction and analysis of models of this type can help us understand the mechanism of the transmission as well as characteristics of diseases, and therefore, we can propose effective strategies to predict, prevent, and restrain diseases, as well as to protect population health. Up to now, many mathematical models for infectious diseases formulated by differential equations have been constructed and analyzed to study the spreading of viruses, for instance, [6–8, 18, 34]. Recently, mathematical models for COVID-19 epidemic have attracted the attention of many mathematicians, biologists, epidemiologists, pharmacists, chemists with many notable and important results (see, for instance, [1, 5, 9, 14, 15, 19, 23, 26, 28–30, 33, 35, 36] and references therein). This can be considered an effective approach to study, simulate, and predict the mechanism and transmission of COVID-19.

Motivated by the above reason, in this work, we formulate and analyze a new mathematical model for COVID-19 epidemic. This model is described by a system of fractional-order differential equations model and includes five classes, namely, *S* (susceptible class), *E* (exposed class), *I* (infected class), *Q* (isolated class), and *R* (recovered class). This model is a generalization of a well-known ODE model formulated in [35]. In the proposed fractional-order model, we use the Caputo fractional derivative instead of the integer-order one because when modeling processes and phenomena arising in the real world, fractional-order models are more accurate than integer-order ones. In particular, fractional-order models provide more degrees of freedom in the model while an unlimited memory is also guaranteed in contrast to integer-models with limited memory [11, 24, 27].

Our main aim is to study the dynamics and numerical approximations for the proposed fractional-order model. Firstly, the positivity and boundedness of the model are investigated based on standard techniques of mathematical analysis. Secondly, the basic reproduction number of the model is calculated by using the next generation matrix approach. Then, asymptotic stability of the model is investigated based on the Lyapunov stability theorem for fractional dynamical systems. Lastly, we apply the adaptive predictor–corrector algorithm and fourth-order Runge–Kutta (RK4) method formulated in [20] to simulate the proposed model. Consequently, a set of numerical simulations is performed to support the validity of the theoretical results. The numerical simulations indicate that there is a good agreement between theoretical results and numerical ones.

This work is organized as follows. The fractional-order differential model is formulated in Sect. 2. Dynamics of the model is investigated in Sect. 3. Numerical simulations by the adaptive predictor–corrector algorithm are performed in Sect. 4. The last section present some remarks, conclusions, and discussions.

## Model formulation

The total population is divided into five compartments: susceptible (*S*), exposed (*E*), infected (*I*), isolated (*Q*), and recovered (*R*) from the disease. We assume that all the classes are normalized. This leads to the mathematical model formulation in which the interaction of the exposed population and infected population is linked to the susceptible population. In this model, we assume that the natural death rate includes the disease death rate. When there is no symptom of the disease, the exposed class moves with a certain rate to the isolated class, but in the case when symptoms are developed, it moves to the infected class. Keeping in mind the above assumptions, we obtain the following ODE model (see [35]):

1 $$ \begin{aligned} &\frac{dS(t)}{dt}= \varLambda -\mu S(t)-\beta S(t) \bigl(E(t)+I(t) \bigr), \\ &\frac{dE(t)}{dt}=\beta S(t) \bigl(E(t)+I(t) \bigr)-\pi E(t)-(\mu + \gamma )E(t), \\ &\frac{dI(t)}{dt}=\pi E(t)-\sigma I(t)-\mu I(t), \\ &\frac{dQ(t)}{dt}=\gamma E(t)+\sigma I(t)-\theta Q(t)-\mu Q(t), \\ &\frac{dR(t)}{dt}=\theta Q(t)-\mu R(t), \end{aligned} $$

where the parameters and variables used are described in Table 1.

**Table 1:** Parameters in the model

Symbols	Description
*S*	Susceptible population
*E*	Exposed population
*I*	Infected Population
*Q*	Isolated population
*R*	Recovered population
*Λ* = *μN*	Recruitment rate
*β*	Rate at which susceptible move to infected and exposed class
*π*	Rate at which exposed population moves to infected one
*γ*	Rate at which exposed people become isolated
*σ*	Rate at which infected people are added to isolated individuals
*θ*	Rate at which isolated persons become recovered
*μ*	Natural death rate plus disease related death rate

Let us introduce the notation

2 $$ N = S + E + I + Q + R. $$

From the ODE system (1), we obtain

$$ \frac{dN}{dt} = \varLambda - \mu N, $$

which implies that the total population in this case we take as constant because $\varLambda =\mu N$.

To include into the model (1) the past history or hereditary properties, we replace the first derivative by the Caputo fractional derivative. More precisely, we propose the following system of fractional differential equations:

3 $$ \begin{aligned} &{}_{0}^{C}D_{t}^{\alpha }S= \varLambda ^{\alpha } - \mu ^{ \alpha } S -\beta ^{\alpha } S (E + I ), \\ &{}_{0}^{C}D_{t}^{\alpha }E= \beta ^{\alpha } S (E + I )- \pi ^{ \alpha } E -\bigl(\mu ^{\alpha } + \gamma ^{\alpha }\bigr)E, \\ &{}_{0}^{C}D_{t}^{\alpha }I= \pi ^{\alpha } E - \sigma ^{\alpha } I - \mu ^{ \alpha } I, \\ &{}_{0}^{C}D_{t}^{\alpha }Q= \gamma ^{\alpha } E + \sigma ^{\alpha } I - \theta ^{\alpha } Q - \mu ^{\alpha } Q, \\ &{}_{0}^{C}D_{t}^{\alpha }R=\theta ^{\alpha } Q(t)-\mu ^{\alpha } R(t), \end{aligned} $$

where $0 < \alpha < 1$, and ${}_{0}^{C}D_{t}^{\alpha }$ denotes the fractional derivative in the Caputo sense. We refer the readers to [2–4, 10, 25] for the definition of the fractional Caputo derivative.

## Dynamics of the fractional-order model

### Positivity and boundedness

Let us denote

$$ \mathbb{R}^{4}_{+} = \bigl\{ (S, E, I, Q)| S, E, I, Q \geq 0 \bigr\} . $$

The following theorem is proved by using a generalized mean value theorem [21, 22] and a fractional comparison principle [16, Lemma 10].

#### Theorem 1

(Positivity and boundedness)

*Let* $(S_{0}, E_{0}, I_{0}, Q_{0})$*be any initial data belonging to* $\mathbb{R}_{+}^{4}$*and* $(S(t), E(t), I(t), Q(t) )$*be the solution corresponding to the initial data*. *Then*, *the set* $\mathbb{R}^{4}_{+}$*is a positively invariant set of the model* (3). *Furthermore*, *we have*

4 $$ \begin{aligned} &\limsup_{t \to \infty }S(t) \leq S_{\infty } := \frac{\varLambda ^{\alpha }}{\mu ^{\alpha }}, \\ &\limsup_{t \to \infty }E(t) \leq E_{\infty } := \frac{\varLambda ^{\alpha }}{\pi ^{\alpha } + \mu ^{\alpha } + \gamma ^{\alpha }}, \\ &\limsup_{t \to \infty }I(t) \leq I_{\infty } := \frac{\pi ^{\alpha }E_{\infty }}{\sigma ^{\alpha } + \mu ^{\alpha }}, \\ &\limsup_{t \to \infty }Q(t) \leq Q_{\infty } := \frac{\gamma ^{\alpha }E_{\infty } + \sigma ^{\alpha }I_{\infty }}{\delta ^{\alpha } + \mu ^{\alpha }}, \\ &\limsup_{t \to \infty }R(t) \leq \frac{\theta ^{\alpha }}{\mu ^{\alpha }}. \end{aligned} $$

#### Proof

First, it is easy to prove the existence and unique of solutions of the model thanks to results proved in [17].

For the model (3), we have

5 $$ \begin{aligned} &{}_{0}^{C}D_{t}^{\alpha }S |_{S = 0}= \varLambda ^{\alpha } > 0, \\ &{}_{0}^{C}D_{t}^{\alpha }E |_{E = 0}= \beta ^{\alpha } SI \geq 0, \\ &{}_{0}^{C}D_{t}^{\alpha }I |_{I = 0}= \pi ^{\alpha } E \geq 0, \\ &{}_{0}^{C}D_{t}^{\alpha }Q |_{Q = 0}= \gamma ^{\alpha } E + \sigma ^{ \alpha } I \geq 0. \end{aligned} $$

By (5) and the generalized mean value theorem [21, 22], we deduce that $S(t), E(t), I(t), Q(t) \geq 0$ for all $t \geq 0$.

From the first equation of the system (3), we obtain

$$ _{0}^{C}D_{t}^{\alpha }S \leq \varLambda ^{\alpha } - \mu ^{\alpha } S. $$

By using the fractional comparison principle, we have the first estimate of (4).

From the second equation of the system (3), we have

$$ _{0}^{C}D_{t}^{\alpha }(S + E) \leq \varLambda ^{\alpha } - \mu ^{\alpha } S - \pi ^{\alpha } E - \bigl(\mu ^{\alpha } + \gamma ^{\alpha }\bigr)E, $$

which implies that

$$ \limsup_{t \to \infty } \bigl(S(t) + E(t) \bigr) \leq E_{\infty }. $$

Consequently, we have the second estimate of (4).

From the third equation of the system (3), we get

$$ _{0}^{C}D_{t}^{\alpha }I \leq \pi ^{\alpha } E_{\infty } - \sigma ^{ \alpha } I - \mu ^{\alpha } I $$

for *t* large enough. This implies the third estimate of (4).

From the fourth equation of the system (3), we have

$$ _{0}^{C}D_{t}^{\alpha }Q \leq \gamma ^{\alpha } E_{\infty } + \sigma ^{ \alpha } I_{\infty } - \theta ^{\alpha } Q - \mu ^{\alpha } Q, $$

for *t* large enough. From this we get the fourth estimate of (4).

Finally, the fifth equation of (3) implies that

$$ _{0}^{C}D_{t}^{\alpha }R \leq \theta ^{\alpha } Q_{\infty } - \mu ^{ \alpha }R, $$

which implies the fifth estimate of (4). The proof is complete. □

### Equilibria and the reproduction number

Firstly, to find equilibria of the model (3), we consider the following algebraic system:

6 $$ \begin{aligned} &\varLambda ^{\alpha } - \mu ^{\alpha } S -\beta ^{\alpha } S (E + I ) = 0, \\ &\beta ^{\alpha } S (E + I )- \pi ^{\alpha } E -\bigl(\mu ^{\alpha } + \gamma ^{\alpha }\bigr)E = 0, \\ &\pi ^{\alpha } E - \sigma ^{\alpha } I - \mu ^{\alpha } I = 0, \\ &\gamma ^{\alpha } E + \sigma ^{\alpha } I - \theta ^{\alpha } Q - \mu ^{ \alpha } Q = 0, \\ &\theta ^{\alpha } Q -\mu ^{\alpha } R = 0. \end{aligned} $$

By some algebraic manipulations, we obtain two solutions of the system (6) that are

7 $$ S_{0} = \frac{\varLambda ^{\alpha }}{\mu ^{\alpha }}, \qquad E_{0} = 0,\qquad I_{0} = 0,\qquad E_{0} = 0,\qquad R_{0} = 0, $$

and

8 $$ \begin{aligned} &S^{*} = \frac{\varLambda ^{\alpha } - (\pi ^{\alpha } + \mu ^{\alpha } + \gamma ^{\alpha })E^{*}}{\mu ^{\alpha }},\qquad I^{*} = \frac{\pi ^{\alpha }}{\sigma ^{\alpha } + \mu ^{\alpha }}E^{*}, \\ & Q^{*} = \frac{\gamma ^{\alpha }E^{*} + \sigma ^{\alpha }I^{*}}{\theta ^{\alpha } + \mu ^{\alpha }}, \\ &E^{*} = \frac{\beta ^{\alpha }\varLambda ^{\alpha }(\pi ^{\alpha } + \sigma ^{\alpha } + \mu ^{\alpha }) - \mu ^{\alpha }(\mu ^{\alpha } + \sigma ^{\alpha })(\pi ^{\alpha } + \mu ^{\alpha } + \gamma ^{\alpha })}{\beta ^{\alpha }(\pi ^{\alpha } + \mu ^{\alpha } + \gamma ^{\alpha })(\pi ^{\alpha } + \sigma ^{\alpha } + \mu ^{\alpha })},\qquad R^{*} = \frac{\theta ^{\alpha }Q^{*}}{\mu ^{\alpha }}. \end{aligned} $$

Note that $S^{*}$, $E^{*}$, $I^{*}$, $Q^{*}$ and $R^{*}$ are positive if and only if

$$ \beta ^{\alpha }\varLambda ^{\alpha }\bigl(\pi ^{\alpha } + \sigma ^{\alpha } + \mu ^{\alpha }\bigr) - \mu ^{\alpha }\bigl( \mu ^{\alpha } + \sigma ^{\alpha }\bigr) \bigl(\pi ^{ \alpha } + \mu ^{\alpha } + \gamma ^{\alpha }\bigr) > 0. $$

We now compute the reproduction number of the model (3) by using the next generation matrix approach developed by van den Driessche and Watmough [31]. Let $x = (E, I, Q, R, S)$. We rewrite the model (3) in the matrix form

9 $$ _{0}^{C}D_{t}^{\alpha }x = \mathcal{F}(x) - \mathcal{V}(x), $$

where

10 $$F(x)=\left(\begin{array}{c} \beta^{\alpha }S(E+I)\\ 0\\ 0\\ 0\\ 0 \end{array}\right),\;V(x)=\left(\begin{array}{c} \pi^{\alpha }E+(\mu^{\alpha }+\gamma^{\alpha })E\\ -\pi^{\alpha }E+\sigma^{\alpha }I+\mu^{\alpha }I\\ -\gamma^{\alpha }E-\sigma^{\alpha }I+\theta^{\alpha }Q+\mu^{\alpha }Q\\ -\theta^{\alpha }Q+\mu^{\alpha }R\\ -\Lambda^{\alpha }+\mu^{\alpha }S+\beta^{\alpha }S(E+I) \end{array}\right).$$

Hence, the reproduction number of the model (3) can be determined by

11 $$ \mathcal{R}_{0} = \rho \bigl(FV^{-1} \bigr) = \frac{\beta ^{\alpha } \varLambda ^{\alpha }(\pi ^{\alpha } + \sigma ^{\alpha } + \mu ^{\alpha })}{\mu ^{\alpha }(\sigma ^{\alpha } + \mu ^{\alpha })(\pi ^{\alpha } + \mu ^{\alpha } +\gamma ^{\alpha })}. $$

It is easy to verify that $S^{*}$, $E^{*}$, $I^{*}$, $Q^{*}$, and $R^{*} > 0$ if and only if $\mathcal{R}_{0} > 1$.

#### Theorem 2

(Equilibria)

*The model* (3) *always possesses a disease*-*free equilibrium* (*DFE*) *point* $F_{0} = (S_{0}, E_{0}, I_{0}, Q_{0}, R_{0})$*given by* (8) *for all values of the parameters*. *Moreover*, *the model has a unique disease endemic equilibrium point* $F^{*} = (S^{*}, E^{*}, I^{*}, Q^{*}, R^{*})$*given by* (9) *if and only if* $\mathcal{R}_{0} > 1$.

### Stability analysis

#### Theorem 3

*The DFE point of the model* (3) *is locally asymptotically stable if* $\mathcal{R}_{0} < 1$.

#### Proof

The Jacobian matrix of the model (3) at the DFE point is

12 $$J(F_{0})=\left(\begin{array}{ccccc} -\mu^{\alpha } & -\beta^{\alpha }S_{0} & -\beta^{\alpha }S_{0} & 0 & 0\\ 0 & \beta^{\alpha }S_{0}-(\pi^{\alpha }+\mu^{\alpha }+\gamma^{\alpha }) & \beta^{\alpha }S_{0} & 0 & 0\\ 0 & \pi^{\alpha } & -(\sigma^{\alpha }+\mu^{\alpha }) & 0 & 0\\ 0 & \gamma^{\alpha } & \sigma^{\alpha } & -(\theta^{\alpha }+\mu^{\alpha }) & 0\\ 0 & 0 & 0 & \theta^{\alpha } & -\mu^{\alpha } \end{array}\right).$$

The characteristic polynomial of *J* is

$$ P_{J}(x) = \bigl(\lambda + \mu ^{\alpha }\bigr) \bigl[\lambda + \bigl(\theta ^{\alpha }+ \mu ^{\alpha }\bigr) \bigr]\bigl(\lambda + \mu ^{\alpha }\bigr) \bigl(\lambda ^{2} + \tau _{1} \lambda + \tau _{2}\bigr), $$

where

13 $$ \begin{aligned} &\tau _{1}= - \bigl[\beta S_{0} - \bigl(\pi ^{\alpha } + \mu ^{ \alpha } + \gamma ^{\alpha }\bigr) - \bigl(\sigma ^{\alpha } + \mu ^{\alpha } \bigr) \bigr], \\ &\tau _{2}= \bigl(\pi ^{\alpha } + \mu ^{\alpha } + \gamma ^{\alpha }\bigr) \bigl( \sigma ^{\alpha } + \mu ^{\alpha }\bigr) - \beta ^{\alpha }S_{0}\bigl(\pi ^{\alpha } + \sigma ^{\alpha } + \mu ^{\alpha }\bigr). \end{aligned} $$

It is easy to verify that if $\mathcal{R}_{0} < 1$, then $\tau _{1} > 0$ and $\tau _{2} > 0$. This implies that two roots of the polynomial $\lambda ^{2} + \tau _{1} \lambda + \tau _{2}$ have negative real parts. Consequently, the real parts of five eigenvalues of the matrix $J(F_{0})$ are all negative, or equivalently, $F_{0}$ is locally stable. The proof is complete. □

We now prove the uniform asymptotical stability of the DFE point of the model (3) by using the Lyapunov stability theorem [12, Theorem 3.1].

#### Theorem 4

*If* $\mathcal{R}_{0} < 1$, *then the DFE point of the model* (3) *is not only locally asymptotically stable but also uniformly asymptotically stable*.

#### Proof

Since the first three equations of the model (3) do not depend on the states *Q* and *R*, we need only consider the following subsystem:

14 $$ \begin{aligned} &{}_{0}^{C}D_{t}^{\alpha }S= \varLambda ^{\alpha } - \mu ^{ \alpha } S -\beta ^{\alpha } S (E + I ), \\ &{}_{0}^{C}D_{t}^{\alpha }E= \beta ^{\alpha } S (E + I )- \pi ^{ \alpha } E -\bigl(\mu ^{\alpha } + \gamma ^{\alpha }\bigr)E, \\ &{}_{0}^{C}D_{t}^{\alpha }I= \pi ^{\alpha } E - \sigma ^{\alpha } I - \mu ^{ \alpha } I. \end{aligned} $$

From (4), it is sufficient to consider the model (3) in its feasible set defined by

$$ \varOmega = \biggl\{ (S, E, I)| S, E, I \geq 0, S \leq \frac{\varLambda ^{\alpha }}{\mu ^{\alpha }} \biggr\} . $$

Consider a Lyapunov function $V: \varOmega \to \mathbb{R}_{+}$ given by

15 $$ V(S, E, I) = \biggl(S - S_{0}\ln \frac{S}{S_{0}} - S_{0} \biggr) + E + \frac{\pi ^{\alpha } + \mu ^{\alpha } + \gamma ^{\alpha }}{\pi ^{\alpha } + \sigma ^{\alpha } + \mu ^{\alpha }}I. $$

By using the linearity property of the Caputo derivative and [32, Lemma 3.1] and some algebraic manipulations, we obtain

$$ _{0}^{C}D_{t}^{\alpha }V \leq -(S - S_{0})^{2} + \tau _{1} I + \tau _{2} E, $$

where

$$ \tau _{1} = \beta ^{\alpha } \frac{\varLambda ^{\alpha }}{\mu ^{\alpha }} - \bigl( \pi ^{\alpha } + \mu ^{\alpha } + \gamma ^{\alpha }\bigr) + \pi ^{\alpha } \frac{\pi ^{\alpha } + \mu ^{\alpha } + \gamma ^{\alpha }}{\pi ^{\alpha } + \sigma ^{\alpha } + \mu ^{\alpha }},\qquad \tau _{2} = \beta ^{\alpha } \frac{\varLambda ^{\alpha }}{\mu ^{\alpha }} - \frac{\pi ^{\alpha } + \sigma ^{\alpha } + \gamma ^{\alpha }}{\pi ^{\alpha } + \sigma ^{\alpha } + \mu ^{\alpha }}. $$

It is easy to check that if $\mathcal{R}_{0} < 1$, then $\tau _{1}, \tau _{2} < 0$. Consequently, by the Lyapunov stability theorem for fractional dynamical systems, we deduce that $F_{0}$ is uniformly asymptotically stable. The proof is complete. □

#### Remark 1

The analysis of stability of $F^{*}$ is an interesting mathematical problem, but in this work, we mainly focus on the case $\mathcal{R}_{0} < 1$ to find an effective strategy to prevent the disease.

### $\mathcal{R}_{0}$ sensitivity analysis

Theorem 3 suggests that we should control the parameters such that $\mathcal{R}_{0} < 1$. This provides a good strategy to prevent and restrain the disease. More precisely, when $\mathcal{R}_{0} < 1$, then

$$ \lim_{t \to \infty } S(t) = \frac{\varLambda ^{\alpha }}{\mu ^{\alpha }}, \qquad \lim _{t \to \infty } E(t) = \lim_{t \to \infty } I(t) = \lim _{t \to \infty } Q(t) = \lim_{t \to \infty } R(t) = 0, $$

which means that the disease will be completely controlled and prevented. Motivated by this, we now perform an $\mathcal{R}_{0}$ sensitivity analysis to find ways to choose suitable parameters.

It is easy to verify that

16 $$ \begin{aligned} &\frac{\partial \mathcal{R}_{0}}{\partial \beta }= \frac{\alpha \beta ^{\alpha - 1}\varLambda ^{\alpha }(\pi ^{\alpha } + \sigma ^{\alpha } + \mu ^{\alpha })}{\mu ^{\alpha }(\sigma ^{\alpha } + \mu ^{\alpha })(\pi ^{\alpha } + \mu ^{\alpha } +\gamma ^{\alpha })} > 0, \\ &\frac{\partial \mathcal{R}_{0}}{\partial \pi }= \frac{\beta ^{\alpha }\varLambda ^{\alpha }}{\mu ^{\alpha }(\sigma ^{\alpha } + \mu ^{\alpha })} \frac{\alpha \pi ^{\alpha - 1}(\gamma ^{\alpha } - \mu ^{\alpha })}{(\pi ^{\alpha } + \mu ^{\alpha } + \gamma ^{\alpha })^{2}}, \\ &\frac{\partial \mathcal{R}_{0}}{\partial \sigma }= - \frac{\beta ^{\alpha }\varLambda ^{\alpha }}{\mu ^{\alpha }(\mu ^{\alpha } + \mu ^{\alpha } + \gamma ^{\alpha })} \frac{\alpha \sigma ^{\alpha -1}\pi ^{\alpha }}{(\sigma ^{\alpha } + \mu ^{\alpha })^{2}} < 0, \\ &\frac{\partial \mathcal{R}_{0}}{\partial \gamma }= - \frac{\beta ^{\alpha }\varLambda ^{\alpha }(\pi ^{\alpha } + \sigma ^{\alpha } + \mu ^{\alpha })}{\mu ^{\alpha }(\sigma ^{\alpha } + \mu ^{\alpha })} \frac{\alpha \gamma ^{\alpha - 1}}{(\pi ^{\alpha } + \mu ^{\alpha } + \gamma ^{\alpha })} < 0. \end{aligned} $$

Equation (16) suggests some ways to choose the parameters such that $\mathcal{R}_{0} < 1$. Hence, based on this, we can propose suitable strategies to control and prevent the disease.

## Numerical simulations by the adaptive predictor–corrector algorithm

### The adaptive predictor–corrector algorithm

In this section we review the method that is proposed by [20]. The proposed algorithm is given as follows. Consider the initial value problem (IVP) governed by:

17 $$ \textstyle\begin{cases} D^{\alpha ,\rho }_{a+}y(t)=f (t,y(t) ), & t\in [0,T], \\ y^{k}(a)=y_{0}^{k},& k=0,1,2,\dots ,[\alpha ]-1, \end{cases} $$

where $D^{\alpha ,\rho }_{a+}$ is the proposed generalized Caputo-type fractional derivative operator given [20, Definition 4]. Initially, for $m-1< \alpha \leq m$, $a\geq 0$, $\rho >0$ and $y\in C^{m} ([a,T])$, the IVP (17) is equivalent, using [20, Theorem 3], to the Volterra integral equation:

18 $$ y(t)=u(t)+\frac{\rho ^{1-\alpha }}{\varGamma (\alpha )} \int ^{t}_{a}(s)^{ \rho -1} \bigl(t^{\rho }-s^{\rho }\bigr)^{\alpha -1}f \bigl(s,y(s) \bigr)\,ds, $$

where

19 $$ u(t)=\sum^{m-1}_{n=0} \frac{1}{\rho ^{n} n!}\bigl(t^{\rho }-a^{\rho } \bigr)^{n} \biggl[ \biggl(x^{1-\rho }\frac{d}{dx} \biggr)^{n}y(x) \biggr]\bigg|_{x=a}. $$

The first step of our algorithm, under the assumption that the function *f* is such that a unique solution exists on some interval $[a,T]$, consists of dividing the interval $[a,T]$ into *N* unequal subintervals $[t_{k},t_{k+1} ]$, $k=0,1,\ldots ,N-1$ using the mesh points

20 $$ \textstyle\begin{cases} t_{0}=a, \\ t_{k+1}= (t_{k}^{\rho }+h )^{\frac{1}{\rho }}& k=0,1,2,\dots ,N-1, \end{cases} $$

where $h=\frac{(T^{\rho }-a^{\rho })}{N}$. Now, we are going to generate the approximations $y_{k}$, $k=0,1,\ldots ,N$, to solve numerically the IVP (17). The basic step, assuming that we have already evaluated the approximations $y_{i}\approx y(t_{j} )$ ($j=1,2,\ldots ,k$), is that we want to get the approximation $y_{k}\approx y(t_{k+1})$ by means of the integral equation

21 $$ y(t_{k+1})=u(t_{k+1})+ \frac{\rho ^{-\alpha }}{\varGamma (\alpha )} \int ^{t_{k+1}}_{a}s^{ \rho -1} \bigl(t_{k+1}^{\rho }-s^{\rho }\bigr)^{\alpha -1}f \bigl(s,y(s) \bigr)\,ds. $$

Making the substitution

22 $$ z=(s)^{\rho }, $$

we get

23 $$ y(t_{k+1})=u(t_{k+1})+ \frac{\rho ^{-\alpha }}{\varGamma (\alpha )} \int ^{t^{\rho }_{k+1}}_{a}\bigl(t_{k+1}^{\rho }-z \bigr)^{\alpha -1}f (z^{\frac{1}{\rho }},y\bigl(z^{ \frac{1}{\rho }} \bigr)\,dz, $$

that is,

24 $$ y(t_{k+1})=u(t_{k+1})+ \frac{\rho ^{-\alpha }}{\varGamma (\alpha )}\sum^{k}_{j=0} \int ^{t^{\rho }_{j +1}}_{t_{j}^{\rho }}\bigl(t_{k+1}^{\rho }-z \bigr)^{\alpha -1}f (z^{\frac{1}{\rho }},y\bigl(z^{\frac{1}{\rho }} \bigr)\,dz. $$

Next, if we use the trapezoidal quadrature rule with respect to the weight function $(t_{k+1}^{\rho }-z)^{\alpha -1}$ to approximate the integrals appear in the right-hand side of Eq. (24), replacing the function $f (z^{\frac{1}{\rho }},y(z^{\frac{1}{\rho }}) )$ by its piecewise linear interpolant with nodes chosen at the $t_{j}^{\rho } $ ($j=0,1,\ldots ,k+1$), then we obtain

25 $$\begin{aligned}& \int ^{t^{\rho }_{j+1}}_{t_{j}^{\rho }}\bigl(t_{k+1}^{\rho }-z \bigr)^{\alpha -1}f (z^{ \frac{1}{\rho }},y\bigl(z^{\frac{1}{\rho }} \bigr)\,dz \\& \quad \approx\frac{h^{\alpha }}{\alpha (\alpha +1)} \bigl[\bigl((k-j)^{\alpha +1}-(k-j- \alpha +) (k-j+1)^{\alpha }\bigr)f \bigl(t_{j},y(t_{j} ) \bigr) \\& \qquad {}+\bigl((k-j+1)^{\alpha +1}-(k-j+\alpha +1) (k-j)^{\alpha } \bigr)f \bigl(t_{j+1},y(t_{j+1} ) \bigr) \bigr]. \end{aligned}$$

Thus, substituting the above approximations into Eq. (24), we obtain the corrector formula for $y(t_{k+1})$, $k=0,1,\ldots ,N-1$,

26 $$ y(t_{k+1})\approx u(t_{k+1})+ \frac{\rho ^{-\alpha }}{\varGamma (\alpha +2)}\sum^{k}_{j=0}a_{j,k+1}f \bigl(t_{j},y(t_{j} ) \bigr)+ \frac{\rho ^{-\alpha }h^{\alpha }}{\varGamma (\alpha +2)}f \bigl(t_{k+1},y(t_{k+1} ) \bigr), $$

where

27 $$ a_{j,k+1}=\textstyle\begin{cases} k^{\alpha +1}-(k-\alpha )(k+1)^{\alpha } \quad \text{if } j=0, \\ (k-j+2)^{\alpha +1}+(k-j)^{\alpha +1}-2(k-j+1)^{\alpha +1}\quad \text{if } 1 \leq j< k. \end{cases} $$

The last step of our algorithm is to replace the quantity $y(t_{k+1})$ shown on the right-hand side of formula (26) with the predictor value $y^{p} (t_{k+1})$ that can be obtained by applying the one-step Adams–Bashforth method to the integral equation (23). In this case, by replacing the function $f (z^{\frac{1}{\rho }},y(z^{\frac{1}{\rho }}) )$ with the quantity $f (t_{j},y(t_{j} ) )$ at each integral in Eq. (24), we get

28 $$\begin{aligned} y^{p}(t_{k+1}) \approx & u(t_{k+1})+ \frac{\rho ^{-\alpha }}{\varGamma (\alpha )}\sum^{k}_{j=0} \int ^{t^{\rho }_{j +1}}_{t_{j}^{\rho }}\bigl(t_{k+1}^{\rho }-z \bigr)^{\alpha -1}f \bigl(t_{j},y(t_{j} ) \bigr)\,dz \\ =&u(t_{k+1})+\frac{\rho ^{-\alpha }h^{\alpha }}{\varGamma (\alpha +1)}\sum^{k}_{j=0} \bigl[(k+1-j)^{\alpha }-(k-j)^{\alpha } \bigr]f \bigl(t_{j},y(t_{j} ) \bigr). \end{aligned}$$

Therefore, the present adaptive predictor–corrector algorithm, for evaluating the approximation $y_{k+1}\approx y(t_{k+1})$, is completely described by the formula

29 $$ y_{k+1}\approx u(t_{k+1})+ \frac{\rho ^{-\alpha }h^{\alpha }}{\varGamma (\alpha +2)}\sum^{k}_{j=0}a_{j,k+1}f (t_{j},y_{j} )+ \frac{\rho ^{-\alpha }h^{\alpha }}{\varGamma (\alpha +2)}f \bigl(t_{k+1},y_{k+1}^{p} \bigr), $$

where $y_{j}\approx y(t_{j} )$, $j=0,1,\ldots ,k$, and the predicted value $y_{k+1}^{p}\approx y^{p} (t_{k+1})$ can be determined as described in Eq. (28) with the weights $a_{j,k+1}$ being defined according to (27). It is easily observed that when $\rho =1$, the present adaptive predictor–corrector algorithm will be reduced to the predictor–corrector approach presented in [13].

### Implementation with numerical simulation

In this section, we solve numerically Eq. (3) using the method given in the former section. In view of the above algorithm, following the rule (27), the approximations $S_{k+1}$, $E_{k+1}$, $I_{k+1}$, $Q_{k+1}$, and $R_{k+1}$ can be simply evaluated using the iterative formulas, for $N\in \mathbb{N}$ and $T>0$,

$$\begin{aligned} &S_{k+1}\approx S_{0}+ \frac{\rho ^{-\alpha }h^{\alpha }}{\varGamma (\alpha +2)}\sum ^{k}_{j=0}a_{j,k+1} \bigl[ \varLambda ^{\alpha }-\mu ^{\alpha } S_{j}-\beta ^{\alpha }S_{j}(E_{j}+I_{j}) \bigr] \\ &\hphantom{S_{k+1}\approx}{}+\frac{\rho ^{-\alpha }h^{\alpha }}{\varGamma (\alpha +2)} \bigl[A^{\alpha }- \mu ^{\alpha }S_{k+1}^{p})- \beta ^{\alpha }S_{k+1}^{p}\bigl(E_{k+1}^{p}+I_{k+1}^{p} \bigr) \bigr], \\ &E_{k+1}\approx E_{0}+ \frac{\rho ^{-\alpha }h^{\alpha }}{\varGamma (\alpha +2)}\sum ^{k}_{j=0}a_{j,k+1} \bigl[\beta ^{\alpha }S_{j}(E_{j}+I_{j})-\bigl( \pi ^{\alpha }+\mu ^{\alpha }+ \gamma ^{\alpha } \bigr)E_{j} \bigr] \\ &\hphantom{E_{k+1}\approx}{}+\frac{\rho ^{-\alpha }h^{\alpha }}{\varGamma (\alpha +2)} \bigl[\beta ^{ \alpha }S_{k+1}^{p} \bigl(E_{k+1}^{p}+I_{k+1}^{p} \bigr)- \bigl(\pi ^{\alpha }+\mu ^{ \alpha }+\gamma ^{\alpha } \bigr)E_{k+1}^{p} \bigr], \\ &I_{k+1}\approx I_{0}+ \frac{\rho ^{-\alpha }h^{\alpha }}{\varGamma (\alpha +2)}\sum ^{k}_{j=0}a_{j,k+1} \bigl[\pi ^{\alpha } E_{j}-\bigl(\sigma ^{\alpha }+\mu ^{\alpha }\bigr)I_{j} \bigr] \\ &\hphantom{I_{k+1}\approx}{}+\frac{\rho ^{-\alpha }h^{\alpha }}{\varGamma (\alpha +2)} \bigl[\pi ^{ \alpha } E_{k+1}^{p}- \bigl(\sigma ^{\alpha }+\mu ^{\alpha }\bigr)I_{k+1}^{p} \bigr], \\ &Q_{k+1}\approx Q_{0}+ \frac{\rho ^{-\alpha }h^{\alpha }}{\varGamma (\alpha +2)}\sum ^{k}_{j=0}a_{j,k+1} \bigl[ \gamma ^{\alpha } E_{j}+\sigma ^{\alpha } I_{j} -\bigl(\theta ^{\alpha }+ \mu ^{\alpha } \bigr)Q_{j} \bigr] \\ &\hphantom{Q_{k+1}\approx}{}+\frac{\rho ^{-\alpha }h^{\alpha }}{\varGamma (\alpha +2)} \bigl[\gamma ^{ \alpha } E_{k+1}^{p}+ \sigma ^{\alpha } I_{k+1}^{p} -\bigl(\theta ^{\alpha }+ \mu ^{\alpha }\bigr)Q_{k+1}^{p} \bigr], \\ &R_{k+1}\approx R_{0}+ \frac{\rho ^{-\alpha }h^{\alpha }}{\varGamma (\alpha +2)}\sum ^{k}_{j=0}a_{j,k+1} \bigl[ \theta ^{\alpha } Q_{j} -\mu ^{\alpha }R_{j} \bigr] \\ &\hphantom{R_{k+1}\approx}{}+\frac{\rho ^{-\alpha }h^{\alpha }}{\varGamma (\alpha +2)} \bigl[\theta ^{ \alpha } Q_{k+1}^{p} -\mu ^{\alpha }R_{k+1}^{p} \bigr], \end{aligned}$$

where $h=\frac{T^{\rho }}{N}$ and

$$\begin{aligned}& S_{k+1}^{p}\approx S_{0}+ \frac{\rho ^{-\alpha }h^{\alpha }}{\varGamma (\alpha +1)}\sum^{k}_{j=0} \bigl((k+1-j)^{\alpha }-(k-j)^{\alpha } \bigr) \bigl[\varLambda ^{\alpha }-\mu ^{ \alpha } S_{j}-\beta ^{\alpha }S_{j}(E_{j}+I_{j}) \bigr], \\& E_{k+1}^{p}\approx E_{0}+ \frac{\rho ^{-\alpha }h^{\alpha }}{\varGamma (\alpha +2)}\sum^{k}_{j=0} \bigl((k+1-j)^{\alpha }-(k-j)^{\alpha } \bigr) \bigl[\beta ^{\alpha }S_{j}(E_{j}+I_{j})-\bigl( \pi ^{\alpha }+\mu ^{\alpha }+\gamma ^{\alpha } \bigr)E_{j} \bigr], \\& I_{k+1}^{p}\approx I_{0}+ \frac{\rho ^{-\alpha }h^{\alpha }}{\varGamma (\alpha +2)}\sum^{k}_{j=0} \bigl((k+1-j)^{\alpha }-(k-j)^{\alpha } \bigr) \bigl[\pi ^{\alpha } E_{j}-\bigl( \sigma ^{\alpha }+\mu ^{\alpha }\bigr)I_{j} \bigr], \\& Q_{k+1}^{p}\approx Q_{0}+ \frac{\rho ^{-\alpha }h^{\alpha }}{\varGamma (\alpha +2)}\sum^{k}_{j=0} \bigl((k+1-j)^{\alpha }-(k-j)^{\alpha } \bigr) \bigl[\gamma ^{\alpha } E_{j}+ \sigma ^{\alpha } I_{j} - \bigl(\theta ^{\alpha }+\mu ^{\alpha }\bigr)Q_{j} \bigr], \\& R_{k+1}^{p}\approx R_{0}+ \frac{\rho ^{-\alpha }h^{\alpha }}{\varGamma (\alpha +2)}\sum^{k}_{j=0} \bigl((k+1-j)^{\alpha }-(k-j)^{\alpha } \bigr) \bigl[\theta ^{\alpha } Q_{j} - \mu ^{\alpha }R_{j} \bigr]. \end{aligned}$$

Parameter values which are calculated for basic time of coronavirus used in numerical example are $\varLambda =0.145$; $\mu =0.000411$; $\beta =0.00038$; $\pi =0.00211$; $\gamma =0.0021$; $\sigma =0.0169$; $\theta =0.0181$, with total population $N = 355$ and initial data $(S_{0},E_{0},I_{0},Q_{0},R_{0} )=(153,55,79,68,20)$.

**Figure 1:**
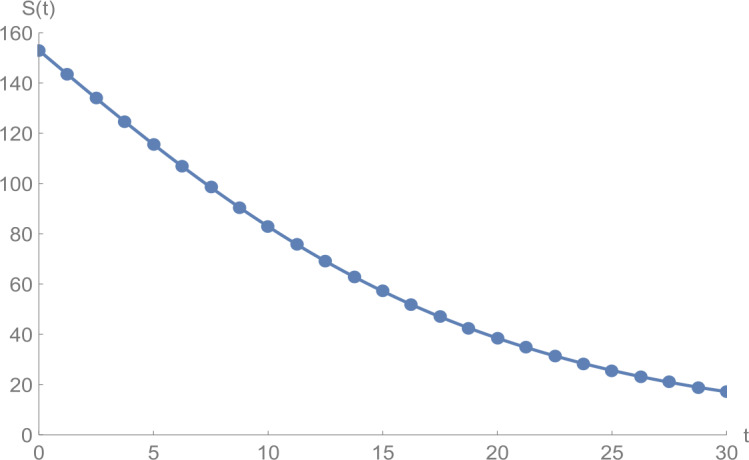
$S(t)$ against *t*: solid line presents the proposed approximation, dotted line stands for RK4 method

**Figure 2:**
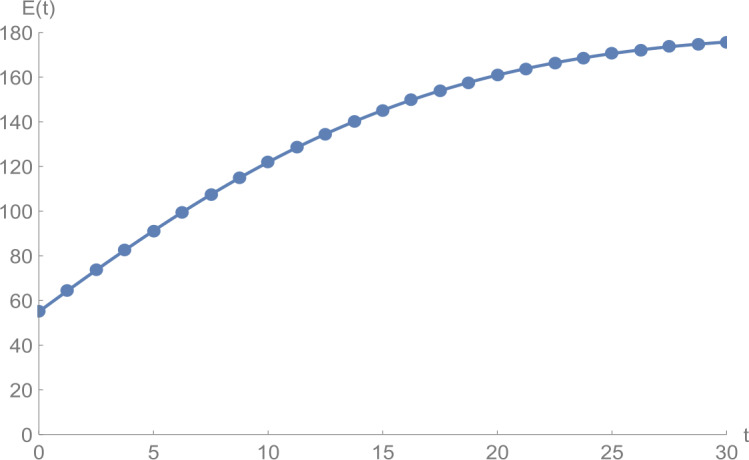
$E(t)$ versus *t*: solid line presents the proposed approximation, dotted line stands for RK4 method

**Figure 3:**
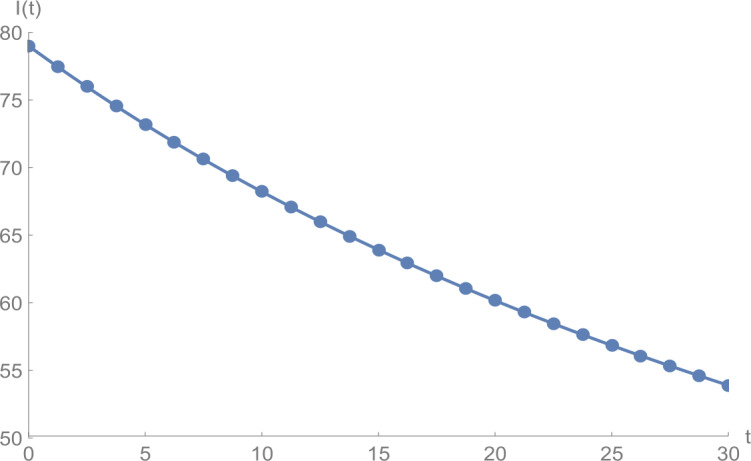
$I(t)$ against *t*: solid line presents the proposed approximation, dotted line stands for RK4 method

**Figure 4:**
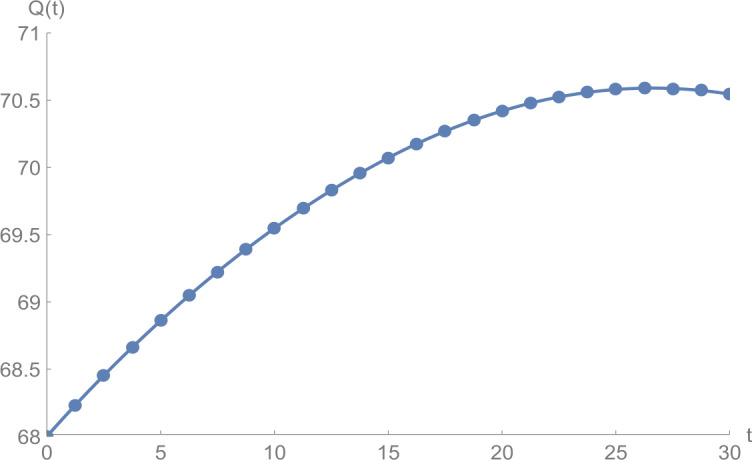
$Q(t)$ against *t*: solid line presents the proposed approximation, dotted line stands for RK4 method

**Figure 5:**
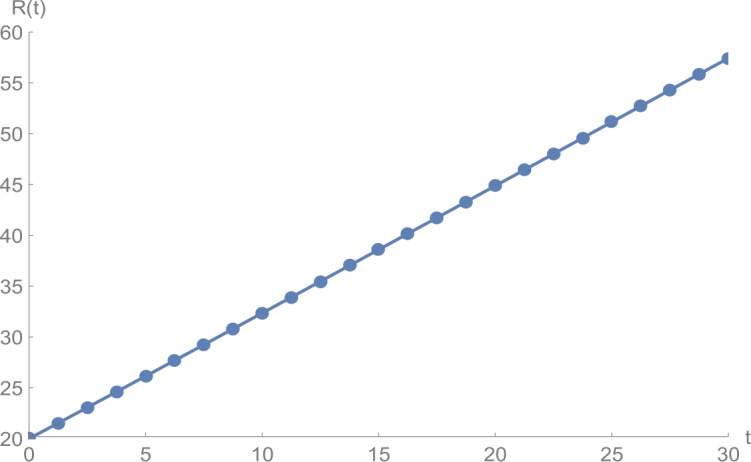
$R(t)$ against *t*: solid line presents the proposed approximation, dotted line stands for RK4 method

In Figs. 1–5, we plot numerical solutions of the model (3) with $T=30$ obtained using the proposed algorithm and the RK4 method when $N=355$ and $(S_{0},E_{0},I_{0},Q_{0},R_{0} )=(153,55,79,68,20)$ for $\alpha =1$. From the graphical results in Figs. 1–5, it can be seen that the results obtained using the proposed algorithm match the results of the RK4 method very well, which implies that the presented method can predict the behavior of these variables accurately in the region under consideration.

**Figure 6:**
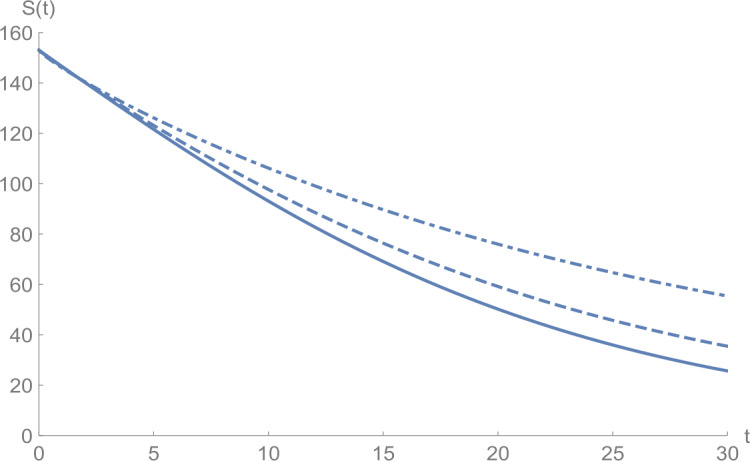
$S(t)$ against *t*: (solid line) $a = 1.0$, (dot-dashed line) $a = 0.85$, (dashed line) $a = 0.75$

**Figure 7:**
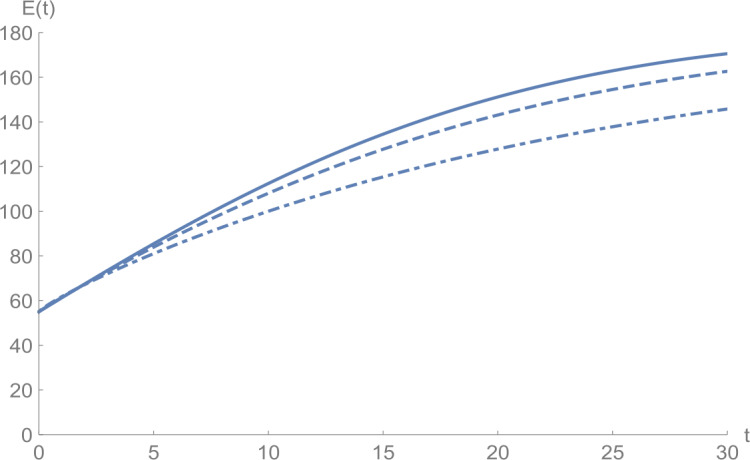
$E(t)$ against *t*: (solid line) $a = 1.0$, (dot-dashed line) $a = 0.85$, (dashed line) $a = 0.75$

**Figure 8:**
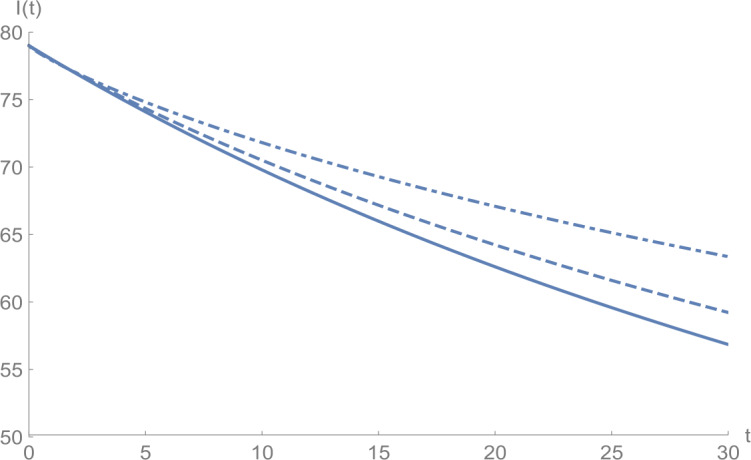
$I(t)$ against *t*: (solid line) $a = 1.0$, (dot-dashed line) $a = 0.85$, (dashed line) $a = 0.75$

**Figure 9:**
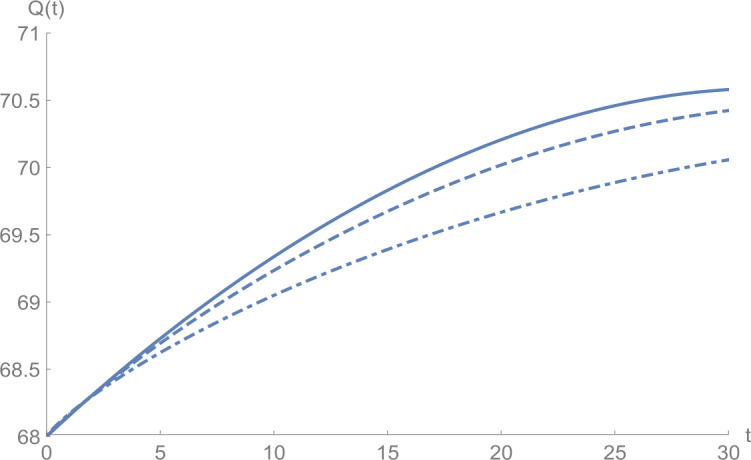
$Q(t)$ against *t*: (solid line) $a = 1.0$, (dot-dashed line) $a = 0.85$, (dashed line) $a = 0.75$

**Figure 10:**
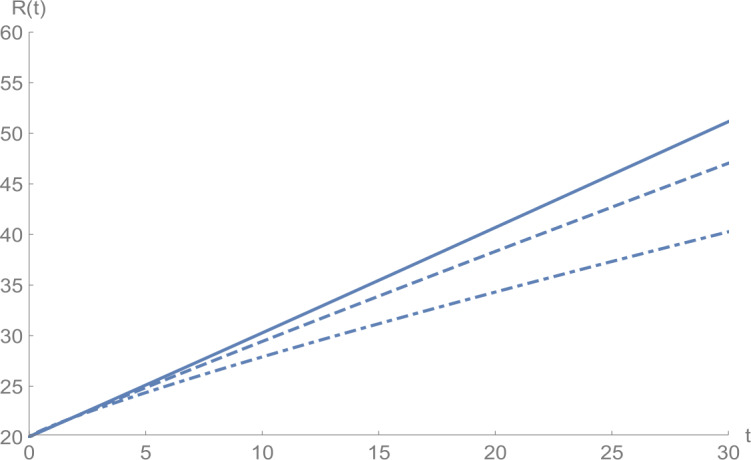
$R(t)$ against *t*: (solid line) $a = 1.0$, (dot-dashed line) $a = 0.85$, (dashed line) $a = 0.75$

Figures 6–10 show the approximate solutions for $S(t)$, $E(t)$, $I(t)$, $Q(t)$, and $R(t)$ obtained for different values of *α* using the proposed algorithm. From the graphical results given in Figs. 6–10, it is clear that the approximate solutions depend continuously on the time-fractional derivative *α*. It is evident that the efficiency of this approach can be dramatically enhanced by decreasing the step size.

## Remarks and conclusions

In this paper, we have formulated and analyzed a new mathematical model for COVID-19 epidemic. The model is described by a system of fractional-order differential equations and includes five classes, namely, *S* (susceptible class), *E* (exposed class), *I* (infected class), *Q* (isolated class), and *R* (recovered class). It should be emphasized that the model is a generalization of our recent work proposed in [35].

Firstly, the positivity, boundedness, and stability of the model have been established. Furthermore, the basic reproduction number of the model has been calculated by using the next generation matrix approach. Lastly, we have applied the adaptive predictor–corrector algorithm and fourth-order Runge–Kutta (RK4) method to simulate the proposed model. A set of numerical simulations has been performed to support the validity of the theoretical results. The numerical simulations indicate that there is a good agreement between theoretical results and numerical ones.

In the near future, we will extend the results in this work to propose new mathematical models for COVID-19 epidemic. Especially, effective strategies to control and prevent the disease will be investigated.
